# Sex-biased mortality associated with inbreeding in *Drosophila melanogaster*

**DOI:** 10.1186/1471-2148-14-51

**Published:** 2014-03-17

**Authors:** Stephen P Robinson, Leigh W Simmons, W Jason Kennington

**Affiliations:** 1 Centre for Evolutionary Biology, School of Animal Biology (M092), The University of Western Australia, Crawley, WA 6009, Australia

## Abstract

**Background:**

One proposed consequence of inbreeding is a skewed sex ratio arising from sex specific mortality in the homogametic sex caused by inbreeding on the sex chromosome. However, recent work suggests that random distortions in sex ratio due to autosomal inbreeding may be of greater importance. In this study, we investigate the effect of biologically realistic levels of inbreeding on sex ratio and sex specific mortality in *Drosophila melanogaster*. We use two pedigree crossing designs to either maximise or minimise inbreeding on the X-chromosome whilst producing identical autosomal inbreeding.

**Results:**

We found increased female mortality and male biased sex ratios associated with inbreeding in our high, but not low, X-inbreeding pedigree. While our results are more consistent with being driven by inbreeding on the X-chromosome than on the autosomes, the marked difference between treatments does not fit closely the expectations of either model.

**Conclusions:**

Our results are only partly consistent with the hypothesis that inbreeding on the X-chromosome can cause greater fitness reductions in the homogametic sex. Whilst the results of our study are not conclusive, they suggest that directional distortions in sex ratio due to inbreeding can occur, and highlight the need for further investigation on this topic.

## Background

Because of their reduced population size and genetic diversity, endangered populations are often vulnerable to inbreeding depression [1]. A proposed consequence of inbreeding, which has received attention due to its implications for endangered populations, is that inbreeding may distort secondary and tertiary sex ratios by increasing the rate of sex specific mortality for the homogametic sex [2-4]. This is of conservation significance because a skewed sex ratio will lead to a substantial reduction in effective population size [5]. For placental mammals and many invertebrates male biased sex ratios are predicted with inbreeding. A male biased sex ratio is of particular concern because extinction risks associated with distortions in sex ratio are not symmetrically distributed around a balanced sex ratio, with male biased sex ratios likely to lead to a much greater risk of extinction [6]. Male biased sex ratios may also lead to further reductions in female fitness due to increased male aggression and competition [7-9]. For example, male biased sex ratios have been linked to increased aggression towards females and have been found to contribute to population collapse in lizards [7]. The directional distortion in sex ratio caused by inbreeding is most comprehensively dealt with by Frankham and Wilcken [4] who give two mechanisms that are expected to contribute to the effect. The first is an increase in the equilibrium frequency of sex linked alleles (those carried on the sex chromosome), which are sex limited (expressed in one sex only), and are deleterious recessive affecting only the homogametic sex. An example of an allele which could produce such an effect is the *Sxl* (Sex lethal) [10] in *Drosophila melanogaster*. As documented by Lindsley and Zimm [11], *Sxl* is a sex linked switch gene that acts throughout development to control all aspects of sexual dimorphism. Its products are required for female development and must be absent for male development. It is common for mutations of this gene to be lethal in homozygous females. Mutations in *Sxl* are largely hidden from selection as they do not affect heterozygous females or hemizygous males, allowing them to accumulate. These deleterious alleles will then be exposed during inbreeding resulting in greater inbreeding depression in females.

The second mechanism relates to dosage compensation [4]. Dosage compensation is a regulatory mechanism in species where the sex chromosomes are highly differentiated that equalises transcription in response to differences in gene dose on the sex chromosomes (see [12] for review). There are a variety of different ways that species achieve dosage compensation. Under some methods of dosage compensation, the recessive alleles that cause sex ratio distortions with inbreeding are not expected to accumulate and therefore directional sex ratio distortions are not expected. Placental mammals usually have random inactivation of one X-chromosome in females. Although a female carrying a deleterious recessive gene on one X-chromosome will express this gene in roughly half of her cells, such females usually have a phenotype closer to the wild type homozygote than the deleterious recessive homozygote (see [4]). Recessive sex linked alleles, which are deleterious only in females, will thus be hidden from selection and able to accumulate. Whereas in marsupials dosage compensation also involves inactivation of one X-chromosome, but it is always the paternally inherited X-chromosome that is inactivated [13]. This means that deleterious recessive sex limited alleles will be exposed to selection when inherited from the maternal side, so that mutations of these alleles will not be able to accumulate on the X. For most insects, birds, and placental mammals, however, some level of directional sex ratio distortion will be expected with inbreeding [4]. Frankham and Wilcken [4] show modelling that suggests that sex-linked alleles (those on the sex chromosome), which are deleterious recessive and expressed equally in both sexes, may also distort sex ratios under inbreeding, leading to a reduction in the homogametic sex in most cases, though again this will not be true in the case of paternal X inactivation.

Empirical results so far do not provide consistent evidence of directional distortions of sex ratio with inbreeding. Although there are numerous examples of inbreeding causing distortions in sex ratio, a meta-analysis by Frankham and Wilcken [4] of 25 vertebrate populations with distorted sex ratios found no evidence of consistent directional distortions. While the authors conclude that, theoretically, a reduction in the homogametic sex is often expected due to inbreeding, random distortions in sex ratio caused by variation in sex limited autosomal loci in small populations are likely to have a larger effect. They suggest that such autosomal sex-limited loci affecting either males or females are likely to be present in approximately equal numbers in large populations, but that in small inbred populations drift may result in unequal numbers affecting each sex.

Frankham and Wilcken [4] suggest that the probability of obtaining a directional distortion of sex-ratio with inbreeding due to sex-linked loci with sex-limited expression is expected to be greater in species with a large proportions of their genome on the sex-chromosome. For this reason they propose that *D. melanogaster,* in which around 20% of the euchromatic genome is on the X-chromosome, is theoretically a likely candidate for a directional distortion in sex ratio. However, studies of this species report mixed evidence. Some studies investigating *D. melanogaster* homozygous for the entire X-chromosome have found evidence for a sex-limited detrimental load on the X-chromosome*,* resulting in a greater inbreeding depression in females e.g. [14-16]. However, Eanes *et al*[17] found no evidence for a sex-limited detrimental load in their study of *D. melanogaster* and highlight some problems with the experimental design of earlier studies. In a recent study by Frankham and Wilcken [4] no evidence for directional distortions in 69 replicate lines of *D. melanogaster* were found after four generations of full sibling mating where the inbreeding coefficient (ƒ) was equal to 0.59. Similarly, a recent study by Kristensen *et al*[18] found no effect of inbreeding on sex ratio in *D. melanogaster* after five generations of full sibling mating (ƒ = 0.67). A factor that may have contributed to the lack of an effect in these two studies is the high level of inbreeding measured, with several generations of full sibling inbreeding occurring prior to assays of sex ratio. This may have caused a reduction in the number of deleterious recessive alleles due to purging [19-21].

Wright’s inbreeding coefficient, ƒ [22] corresponds to the probability that both copies of a gene at a particular autosomal locus are identical through common ancestry. However, the inheritance of the X-chromosome is somewhat different than the autosomes. Males inherit only one copy of the X-chromosome and this must come from their mother. When males reproduce they will always pass on this same X-chromosome to their female offspring and no X-chromosome to their male offspring. This means that depending on whether the common ancestors are male or female, the chance that both of a female’s copies of a gene at a particular locus on the X-chromosome are identical by descent can be much greater or much lower than ƒ.

**Figure 1:**
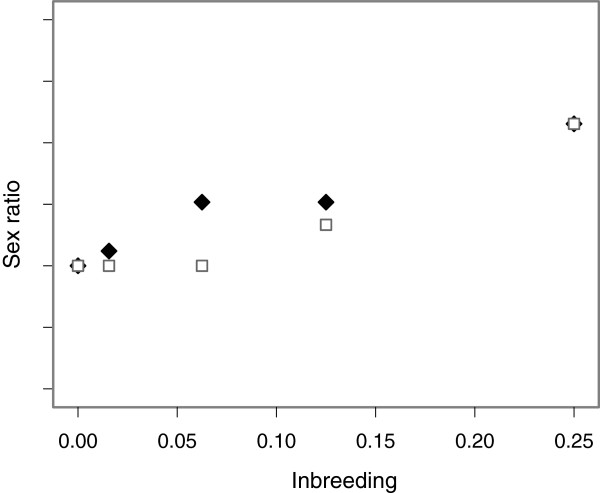
**Predicted sex ratio.** Predicted relationship between sex ratio and autosomal inbreeding coefficient in the two pedigrees. The high X–inbreeding pedigree is shown as black diamonds. The low X-inbreeding pedigree is shown as white squares.

In this study, we investigate the effect of inbreeding on tertiary sex ratio (the sex ratio of sexually mature adults) in *Drosophila melanogaster* using two separate pedigree crossing designs that differ in the level of inbreeding they generate on the X-chromosome. To avoid purging, we consider more biologically realistic levels of inbreeding*,* ranging from unrelated to full sibling crosses (ƒ = 0.25). Figure 1 shows the relative change in sex ratio due to inbreeding on the X-chromosome that we expect to see across the different levels of autosomal inbreeding in each pedigree. Finally, because inbreeding depression is thought to be more severe in stressful environments [23], we investigated the effect of inbreeding on mortality after extreme cold exposure in male and female offspring from our two pedigrees to expose possibly hidden effects of inbreeding not expressed in the relatively benign conditions in the laboratory.

## Results

### The effect of inbreeding on sex ratio

**Figure 2:**
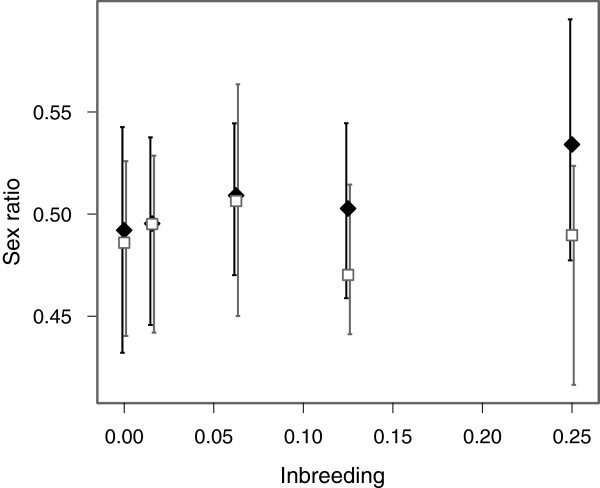
**Sex ratio.** Variation in sex ratio between the two pedigrees plotted against autosomal inbreeding coefficient. The high X-inbreeding pedigree is shown as black diamonds with black error bars. The low X-inbreeding pedigree is shown as white squares with grey error bars. Data has not been corrected for block. Error bars indicate 95% confidence intervals.

In total our sex ratio assay recorded the sex of 31,779 offspring. We found a significant interaction between pedigree (high or low X-inbreeding) and autosomal inbreeding coefficient (χ^2^ = 5.652, df = 1, *P =* 0.017) as well as an effect of pedigree (χ^2^ = 8.552, df = 1, *P* = 0.003). The effect of autosomal inbreeding coefficient was nonsignificant (χ^2^ = 2.826, df = 1, *P = 0.093*). Analysing the results from each pedigree separately, we found that there was a significant effect of autosomal inbreeding coefficient on sex ratio in the high X-inbreeding pedigree (χ^2^ = 7.047, df = 1, *P* = 0.008). Figure 2 shows that the sex ratio became increasingly male biased as the levels of inbreeding increased. However, for the low X-inbreeding pedigree there was no significant effect of inbreeding on sex ratio (χ^2^ = 0.344, df = 1, *P* = 0.558).

Our direct test of inbreeding on the X-chromosome, after controlling for autosomal inbreeding, showed no significant effect on sex ratio beyond that which could be attributed to autosomal inbreeding (F = 2.52, df = 1, *P* = 0.145). Simulations to assess the statistical power of this test revealed that we could detect a significant X-chromosome inbreeding effect in just 46% of cases when there was a 10% change in sex ratio due to inbreeding on the X-chromosome in full sib crosses. When the linear effect of inbreeding on the X-chromosome was increased to 20%, a significant X-chromosome inbreeding effect was found in 96% of cases. A significant effect of autosomal inbreeding was found in less than 6% of simulations in either scenario after inbreeding on the X-chromosome was taken into account.

Analysis of QAIC values indicated that a model including block and inbreeding on the X-chromosome was most supported by our data, with a QAIC weight twice as large as that of the next best model (Table 1).

**Table 1:** Results of the two separate AIC model selection analyses on quasibinomial models explaining variation in sex ratio and female cold shock mortality respectively

**Response**	**Model**	**Factors**	**K**	**QAIC**	**QAIC weight**	**Quasi log likelihood**
Sex ratio	1	Block + X chromosome inbreeding	12	615.21	0.52	-295.60
	2	Block + autosomal inbreeding	12	616.55	0.26	-296.28
	3	Block	11	616.94	0.22	-297.47
Female cold shock mortality	1	Block + X chromosome inbreeding	12	189.83	0.54	-82.92
	2	Block + autosomal inbreeding	12	191.26	0.27	-83.63
	3	Block	11	191.90	0.19	-84.95

Within each AIC selection analysis a lower QAIC value indicates a model with a better fit and the QAIC weight represent the relative likelihood of the model.

### The effect of inbreeding on cold shock mortality

**Figure 3:**
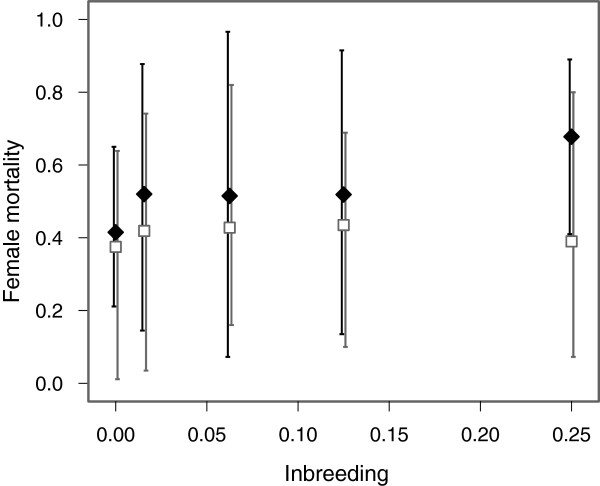
**Female mortality.** Variation in female mortality between the two pedigrees plotted against autosomal inbreeding coefficient. The high X-inbreeding pedigree is shown as black diamonds with black error bars. The low X-inbreeding pedigree is shown as white squares with grey error bars. Data has not been corrected for block. Error bars indicate 95% confidence intervals.

For female cold shock mortality we found a similar pattern to that on sex ratio, with a significant pedigree type by autosomal inbreeding coefficient interaction (χ^2^ = 9.079, df = 1, *P* = 0.003) as well as a significant effect of pedigree type (χ^2^ = 4.368, df = 1, *P* = 0.037). There was a marginally nonsignificant effect of autosomal inbreeding coefficient (χ^2^ = 3.561, df = 1, *P* = 0.059). Figure 3 shows that the interaction between pedigree type and autosomal inbreeding coefficient is similar to that found for sex ratio, with female mortality increasing with increased inbreeding in the high X-inbreeding pedigree, but no effect of inbreeding on female mortality in the low X-inbreeding pedigree. Analysing the high X-inbreeding and low X-inbreeding pedigrees separately we found a significant effect of inbreeding in the high X-inbreeding pedigree (χ^2^ = 6.628, df = 1, *P* = 0.010), but no effect of inbreeding in the low X-inbreeding pedigree (χ^2^ = 0.001, df = 1, *P* = 0.971). Directly testing for an effect of inbreeding on the X-chromosome after accounting for autosomal inbreeding, again, showed no significant effect (F = 2.107, df = 1, *P* = 0.150). However, analysis of QAIC values indicated that a model including block and inbreeding on the X-chromosome was most supported by our data, with a QAIC weight almost twice that of the next best model (Table 1).

**Figure 4:**
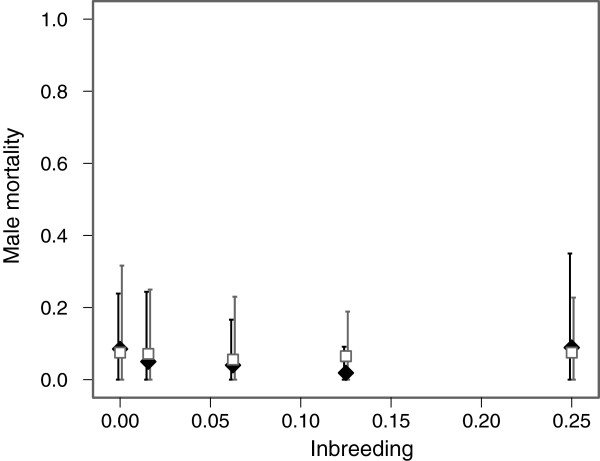
**Male mortality.** Variation in male mortality between the two pedigrees plotted against autosomal inbreeding coefficient. The high X-inbreeding pedigree is shown as black diamonds with black error bars. The low X-inbreeding pedigree is shown as white squares with grey error bars. Data has not been corrected for block. Error bars indicate 95% confidence intervals.

For male cold shock mortality, all of our analyses showed no significant effect of pedigree type (F = 0.425, df = 1, *P* = 0.514), inbreeding coefficient (F = 0.169, df = 1, *P* = 0.681) or their interaction (F = 0.203, df = 1, *P* = 0.652) (Figure 4).

## Discussion

Our results show a male bias in adult sex ratio associated with increased inbreeding in our high X-inbreeding pedigree and no evidence for a distortion in sex ratio in our low X-inbreeding pedigree. These results partially support the hypothesis that sex ratio distortions associated with inbreeding may arise due to deleterious mutations residing on the X-chromosome. However, as can be seen in Figure 1, we expected to see a similar but slightly weaker effect of inbreeding on sex ratio in the low X-inbreeding pedigree. The difference observed between the two pedigrees for full sibling crosses was not predicted due to inbreeding on the X-chromosome or on the autosomes. This is where we observed the largest difference in sex ratios between the two pedigrees, yet for this cross, the level of inbreeding on both the X-chromosome and on the autosomes was identical and cannot account for this difference. Our cold shock mortality results are consistent with the sex ratio distortion being driven by an increase in female mortality. Female mortality increased with the level of inbreeding in the high X-inbreeding pedigree but not in the low X-inbreeding pedigree. Mortality rates after cold shock were far lower in males and we did not see a significant difference in male mortality between pedigrees. However, again, we found the largest difference in female mortality for full sibling crosses where there was no difference between the two pedigrees in either autosomal or X chrosmosome inbreeding. We were also unable to demonstrate directly that increased inbreeding on the X-chromosome, and not the autosomes, was responsible for the skew in sex ratio and the increased cold shock mortality we observed in females. Because autosomal and X-linked inbreeding were correlated in many of our crosses, we lacked power to detect an effect of inbreeding on the X-chromosome after accounting for autosomal inbreeding. Our simulations indicated that for an effect size of 10% difference in sex ratio between unrelated and full sibling crosses, we would expect to detect a significant result in only 46% of cases. Our observed change in sex ratio was closer to 5%. Using a greater number of crosses and a larger difference in X-chromosome inbreeding between the high and low X-inbreeding pedigrees may have increased our power to detect a result. However, this would be difficult to achieve in a single generation. For example, whilst parent offspring crosses could have generated greater levels of inbreeding on the X-chromosome at autosomal inbreeding of ƒ = 0.25, this would have resulted in male and female parents being of different ages between the two pedigrees. Priest *et al*[24] found that increases in either maternal or paternal age were associated with reduced fitness in the offspring of *D. melanogaster,* but that maternal age had the stronger effect. Maternal age has also been found to be directly associated with increased inbreeding depression in other species, for example the seed beetle, *Callosobruchus maculatus*[25].

After accounting for a possible effect of autosomal inbreeding, we were not able to demonstrate a further effect of inbreeding on the X-chromosome. However, in a large outbred population, deleterious recessive sex limited alleles are expected to be present in relatively equal proportions and are therefore not expected to lead to distortions in sex ratio [4]. Our experimental population was established from one hundred and fifty wild-caught, inseminated females and was maintained at high population densities. This should have captured and maintained a large amount of genetic diversity. Whilst it is possible that some of this diversity was lost in the 11 months between the population being established and the experiment being conducted, we would still expect autosomal sex limited deleterious recessives to have been present in relatively equal numbers, and therefore not be responsible for the observed distortion in sex ratio. The results of our AIC model selection analysis also suggest that the skew in sex ratio and female cold shock mortality was more consistent with inbreeding on the X-chromosome than with autosomal inbreeding, although neither model can explain the difference we observed between the pedigrees. It is possible that some other unexplained variable may have differed between the pedigrees resulting in the difference in the response of sex ratio to inbreeding.

Although theoretical work by Frankham and Wilcken [4] predicts some directional distortion in sex ratio due to inbreeding in *D. melanogaster*, neither they nor Kristensen *et al*[18] found evidence of this. As outlined above, a possible explanation for their lack of findings is that several generations of full sibling inbreeding resulted in purging of deleterious recessive alleles before sex ratio was assayed. The population used by Frankham and Wilcken [4] had also been maintained in lab culture for ten years before their experiment. This may have resulted in loss of genetic diversity and is also likely to have caused the flies to become “laboratory adapted” to the food and environment. Inbreeding depression has been shown to be more severe in stressful conditions [23] so the relatively benign conditions in the laboratory may have contributed to a weaker effect.

Our results, which suggest that in *D. melanogaster* females may suffer greater inbreeding depression than males, are somewhat in contrast to the findings of a recent study by Enders and Nunney [26], who found male fitness to be more sensitive to inbreeding depression than female fitness. However, a likely explanation for this difference is that Enders and Nunney [26] considered sexual selection in their fitness measures, whereas we did not. Sexual selection is likely to be stronger on males than females, so this is likely to amplify the effects of inbreeding depression in males. In their study, inbreeding depression was also scored on different traits for males and females, mating success for males and fecundity for females; our results are therefore not directly comparable.

The distortion in sex ratio we observed for full sibling crosses in our high X-inbreeding pedigree was greater than the theoretical predictions for *Drosophila* made by Frankham and Wilcken [4]. They suggest that directional skews in sex ratio may result from a combination of deleterious recessive genes on the X-chromosome that are expressed only in females or in both sexes. They expressed their predictions for directional skews in sex ratio as the sex ratio from full sibling inbreeding divided by the sex ratio from outbred crosses. Their estimates varied depending on the strength of selection against deleterious recessives, but ranged to a maximum of 1.023 (inbred/outbred) for sex linked deleterious recessives expressed in both sexes and 1.015 (inbred/outbred) due to sex linked deleterious recessives expressed in females only. In our high X-inbreeding pedigree we found a sex ratio associated with full sibling crosses substantially higher at 1.190 (inbred/outbred). Frankham and Wilcken’s [4] theoretical work suggests that for invertebrates, autosomal loci are expected to lead to skews in sex ratio of 1.073 (inbred/outbred) for those expressed only in females and 0.932 (inbred/outbred) for those expressed only in males. It is possible that the X-linked effects in our study were greater than predicted by Frankham and Wilcken [4] or that autosomal sex limited deleterious recessive alleles were contributing to this result despite this population being likely to have retained a reasonable amount of genetic diversity.

Although greater than expected, the distortion in sex ratio for *D. melanogaster* was still modest. Assuming that inbreeding on the X-chromosome was driving our results, it should be kept in mind that this species is expected to show a greater effect than most due to a large proportion of its genome being located on the X-chromosome. Nonetheless, because distortions in sex ratio are expected to lead to male biased sex ratios in placental mammals and many insects [4], this would make them more prone to extinction than would purely random distortions in sex ratio. Therefore, the ubiquity of directional distortions in sex ratio warrants further investigation.

## Conclusions

The increased female mortality and male biased sex ratios observed with increased inbreeding in our high X-inbreeding pedigree are consistent with a directional distortion in sex ratio caused by inbreeding on the X-chromosome. However, we were unable to unequivocally rule out inbreeding on the autosomes as a potential contributor to this effect. The marked differences observed between the high and low X-inbreeding pedigrees were not predicted due to inbreeding on the X-chromosome or the autosomes. Nevertheless, the observed results were at least consistent with predictions based on X-chromosome inbreeding. Our results suggest that directional distortions in sex ratio may arise following inbreeding on the X-chromosome and highlight the need for further research on this topic.

## Methods

### Generation of experimental flies

The *Drosophila* stock used in our experiments was established from 151 wild-caught females collected from a single site in Margaret River (located on the south west coast of Western Australia, 33.95 °S, 115.07 °E) in May 2009 and maintained in the laboratory for approximately 11 months (approximately 24 generations with effective population sizes > 200) prior to use in this experiment. The population was maintained with overlapping generations in a single 9 L container on standard agar-maize-yeast medium at 25°C with a 12/12 h light/dark cycle.

**Figure 5:**
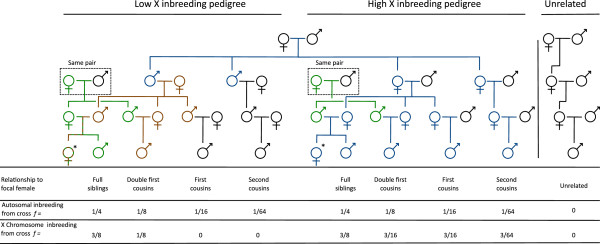
**Pedigree crossing design.** All individuals without origins within the pedigrees were sourced from the mass population. Females marked with a * indicate the focal female group in each pedigree, with which males were crossed to produce the desired levels of inbreeding. The blue, green and brown colourings trace the inheritance of X-chromosomes that may be shared between the focal females and their mates. Same pair indicates that this same pair of individuals was shared in common between the two pedigrees.

From this stock population we used two different pedigrees, each of which generated inbreeding levels equal to crosses between: full siblings (ƒ = 1/4), double 1^st^ cousins (ƒ = 1/8), 1^st^ cousins (ƒ = 1/16), 2^nd^ cousins (ƒ = 1/64) and unrelated individuals (ƒ ≈ 0). Although each pedigree generated identical levels of inbreeding on the autosomes, by altering the sex of the individuals chosen to continue each step in the pedigree, one pedigree was set up to maximise inbreeding on the X-chromosome while the other was set up to minimise inbreeding on the X-chromosome (Figure 5). These will be referred to as the high X-inbreeding and the low X-inbreeding pedigrees respectively. Inbreeding on the X-chromosome was calculated following Crow and Kimura [27] and is listed for each cross within each pedigree in Figure 5. It should be noted that while the low X-inbreeding pedigree aimed to minimise inbreeding on the X-chromosome, this was not always possible and in the case of double first cousin and full sibling crosses, inbreeding on the X-chromosome could not be avoided (see Figure 5). For each level of autosomal inbreeding within each pedigree we also calculated the relative differences in sex ratio expected due to the differences in X-chromosome inbreeding (Figure 1).

To conduct each cross, flies were housed in individual 50 ml vials on 10 ml agar-maize-yeast medium, seeded with 5 mg of live yeast. All virgin flies used in the experiment were collected within seven hours of emerging (before they reached sexual maturity [28]) under brief CO_2_ exposure, and sorted into single sex vials. Virgins were kept for three days prior to use, and these vials were checked for larvae production. Unrelated individuals used in the pedigree where collected by placing vials directly into the mass population cage overnight, and collecting virgin males and females from these vials after 11-12 days. Our experimental design was replicated in 10 separate blocks, each established one day apart. Within each block we generated one high X-inbreeding and one low X-inbreeding pedigree. The pedigrees within a block shared a number of individuals in common. Both were established from the same founding pair of individuals as shown in Figure 5. Another pair of individuals was also shared in common between the blocks used in the second generation in the pedigrees as noted in Figure 5. Males for the unrelated cross for both pedigrees were also sourced from the same family. For each step in the pedigree, males and females were paired in individual vials and allowed to lay eggs for 48 hrs before being transferred to a second laying vial and then discarded after another 48 hrs. Virgin males and females were collected from these two vials after 11 – 12 days as required to establish the next crosses for the pedigree. To ensure that the pedigree was complete even if some crosses failed to produce offspring, four replicates of each step were established and of these, one was chosen at random to continue the pedigree. Each pedigree produced females from a single family (which will be referred to as focal females) that could then be crossed to males of the five different levels of relatedness to them as listed above. For each pedigree, 25 virgin focal females were collected and five replicate crosses of each of the five levels of inbreeding were made. The unrelated males used for this final cross were collected from a vial set up as a replicate of a “full sibling” vial from another block. In this way these unrelated males had gone through the exact same handling as the full sibling males. In total, across all 10 blocks we produced 500 crosses, the offspring of which were collected to measure sex ratio and cold shock mortality. To collect these offspring each cross was allowed to lay eggs sequentially on three laying vials, spending forty-eight hours on each vial. Offspring from the first two vials were collected for the cold shock mortality assay on days 11 and 12 from when the initial cross was established. The offspring from vial three were collected and sexed sixteen days from when females were allowed to commence laying in this vial. To ensure that inbreeding was not distorting sex ratio by delaying development time, which might lead to fewer offspring of one sex emerging before the 16 day collection cut-off, we collected and sexed all offspring from each full sibling cross every day for twenty-one days in two of our blocks. Out of a total of 1212 individuals emerging from these vials, just 22 (1.8%) emerged more than sixteen days after laying commenced. Of these, 8 were female and 14 were male, suggesting that excluding such individuals from our sample is unlikely to generate male biased sex ratios.

### Cold shock mortality

All flies used in this assay were between two and three days old at the time of cold exposure. One replicate of each cross within each pedigree was chosen at random to source offspring for the assay of cold shock mortality. Twenty offspring of each sex from each cross were sorted into single sex laying vials under brief CO_2_ exposure the day before the assay. Cold shock mortality was assessed using the protocols established in previous studies e.g. [29,30]. On the day of the assay each group of 20 flies was transferred to a separate empty glass vial, and the vial partially submerged in a salt water/ice bath at –2°C for three hours. Immediately after cold exposure each group of 20 flies was transferred back to a room temperature laying vial and returned to the controlled temperature room at 25°C. We recorded the number of live and dead flies in each vial five days after cold exposure.

### Statistical analyses

All statistical analyses were carried out in the statistical package R [31]. For sex ratio data, the five replicates of each level of relatedness within each pedigree in each block were pooled prior to analysis by summing the number of adult offspring of each sex. In the rare case where less than two of the five replicates produced viable offspring, all replicates were excluded from the analysis. Analyses of sex ratio, male cold shock mortality and female cold shock mortality were carried out separately.

First, to test whether sex ratio was affected by inbreeding differently in the two pedigrees, data were fitted with a binomial generalized linear model using the “glm” function of the “stats” package. For sex ratio we used the number of male offspring as the number of successes and the number of female offspring as the number of failures. Mortality after cold shock was analysed in a similar manner, using the numbers of live and dead flies after the assay. In all cases response variables were overdispersed, so we fitted the models using quasibinomial error distributions. For each trait, we included the predictor variables block, autosomal inbreeding (ƒ), pedigree type and the interaction between autosomal inbreeding and pedigree type. *P-*values were obtained using the “Anova” function of the “car” package [32] with type II sums of squares (main effects tested without removing variation attributed to interaction terms). Where there was a significant interaction, we separated the data into pedigree type and carried out new analyses with block and autosomal inbreeding as the predictor variables only.

Next, we tested directly for an effect of inbreeding on the X-chromosome after controlling for possible effects of autosomal inbreeding. We analysed the results from both pedigrees simultaneously using a model with predictor variables added in the order block, autosomal inbreeding and X-chromosome inbreeding. Significance testing was carried out using the “anova” command in the “stats” package, which fits model terms sequentially (type I sums of squares). Because the level of autosomal inbreeding and X-chromosome inbreeding were positively correlated (r = 0.94), we may have lacked power to detect an effect of X-chromosome inbreeding after controlling for autosomal inbreeding. In order to test our power to detect such a result, we used simulations to create data sets with a linear distortion in sex ratio for all crosses at a rate equivalent to a 10% difference in sex ratio between unrelated and full sibling crosses driven by inbreeding on the X-chromosome and a variance in sex ratio equal to that which we observed in our actual data. We created 1000 such simulated data sets and calculated the proportion of these where we were able to detect a significant effect using the sample sizes in our study.

Finally, where our initial analysis indicated a significant interaction between inbreeding and pedigree type, we used AIC selection to assess whether any effect was better explained by inbreeding on the X-chromosome or by autosomal inbreeding. AIC selection is useful in allowing for selection between models but does not give any information on goodness-of-fit of the models in question [33]. We used the “aictab” command of the AICcmodavg package [34] to calculate Quasi AIC and Quasi Akaike weights for the binomial models fitted to data from both pedigrees. We included three models for comparison, one with block and X-chromosome inbreeding as factors, one with block and autosomal inbreeding as factors and one with only block as a factor. We used the same overdispersion parameter for each model, calculated from the autosomal inbreeding model using the “c_hat” command of the “AICcmodavg” package.

## Competing interests

The authors declare that they have no competing interests.

## Authors’ contributions

SPR participated in the design of the study, performed the majority of the laboratory work, performed the statistical analysis and drafted the manuscript. WJK participated in the design of the study, assisted in statistical analysis and helped to draft the manuscript. LWS participated in the design of the study, assisted with laboratory work, assisted in statistical analysis and helped to draft the manuscript. All authors read and approved the final manuscript.
